# SOCIAL HEALTH AND IMMUNE SYSTEM IMBALANCE: SEX-SPECIFIC ASSOCIATIONS AND CAUSAL LINKS TO BRAIN AGING

**DOI:** 10.1093/geroni/igac059.1707

**Published:** 2022-12-20

**Authors:** Isabelle van der Velpen, Amber Yaqub, Meike Vernooij, Marieke Perry, Myrra Vernooij-Dassen, Mohsen Ghanbari, Arfan Ikram, Rene Melis

**Affiliations:** Erasmus MC, Rotterdam, Zuid-Holland, Netherlands; Erasmus MC, Rotterdam, Zuid-Holland, Netherlands; Erasmus MC, Rotterdam, Zuid-Holland, Netherlands; Radboud University, Nijmegen, Gelderland, Netherlands; Radboud University, Nijmegen, Gelderland, Netherlands; Erasmus MC, Rotterdam, Zuid-Holland, Netherlands; Erasmus MC, Rotterdam, Zuid-Holland, Netherlands; Radboud university medical center, NIjmegen, Gelderland, Netherlands

## Abstract

**Background:**

We explored whether the balance between innate and adaptive immune system links social health to cognitive brain aging in community-dwelling older adults.

**Methods:**

Social health markers (social support, marital status, loneliness) were measured in the Rotterdam Study in 2002-2008. Balance of the immune system was assessed using white blood-cell-based indices (neutrophil-to-lymphocyte ratio (NLR), platelet-to-lymphocyte ratio (PLR), systemic immune-inflammation index (SII)) during the same visit. Cognitive function and total brain volume were measured at the 2009-2014 follow-up visit.

**Results:**

In 8375 adults (mean age 65.7, 57% female), never married participants had higher NLR, PLR and SII compared to married peers, indicating imbalance towards innate immunity. Widowed/divorced males, but not females, had higher NLR, PLR and SII. Immune system balance did not mediate associations between social health and cognitive brain aging. Discussion: Social health is sex-differentially associated with immune system balance, but does not link to cognitive brain aging through mediation.

